# Public Health and Project Management: Do Projects Deliver?

**DOI:** 10.3390/ijerph17197244

**Published:** 2020-10-03

**Authors:** Farida Saleem, Imran Murtaza, Shabir Hyder, Muhammad Imran Malik

**Affiliations:** 1 Department of Management, Collage of Business Administration, Prince Sultan University, Riyadh 11586, Saudi Arabia; 2 Department of Management Sciences, COMSATS University Islamabad, Attock Campus, Attock 43600, Pakistan; i4imranmurtaza@gmail.com (I.M.); hydershabir@ciit-attock.edu.pk (S.H.); im4imranmalik@gmail.com (M.I.M.)

**Keywords:** maternal newborn and child health, project management, sustainable development goals, public health

## Abstract

Maternal, newborn, and child health (MNCH) has remained an ever-concerning area for hospital management and researchers throughout the world. Nevertheless, in the literature, less attention is paid to developing countries. The current study identifies the problems faced by maternal newborn and child health projects at each phase. We obtained data on MNCH projects via interviews from district project managers and extracted various themes for each phase of the MNCH project. The results indicated the most significant problems faced by the MNCH project emanate from the inefficient bureaucratic structure, lack of realistic planning, weak working environment, political interference, and inefficient knowledge acquisition. The current study found that project managers experience various problems from the initiation stage of the project to its closure. Additionally, they find themselves to be poorly equipped to manage such problems. We proposed various strategies such as implementing a bottom-up management approach, more decentralization, establishing patient feedback systems, giving more authority to the project managers, and so forth.

## 1. Introduction

Over time, there has been remarkable progress in meeting the fourth and fifth Millennium Development Goals (MDGs) [1] related to infant mortality and maternal mortality, respectively [2]. Evidence suggests that the mortality rate of children under five years of age has been reduced by more the half from about 12.7 million to about 6 million during a twenty-five year period (1990–2015) and, similarly, the maternal mortality rate has been reduced across the world by 45 percent [3]. However, most of the success in mortality reduction has been achieved in developed countries, while developing countries are still lagging behind; for example, the risk of maternal death in developed countries is 1 in 3300 as compared to 1 in 41 in developing countries [4,5]. One of the significant factors in reducing the mortality rate is the maternal, newborn, and child health (MNCH) program. Therefore, there is a need to review the MNCH program afresh in developing countries.

Besides medical aspects, the existing literature indicates various social determinants that directly affect the progress towards the improvement of MNCH conditions. It includes poor nutrition, low status of women in society, high poverty, low literacy rates, poor behavior towards health care, poor health facilities, and so forth. Similarly, women’s empowerment, high fertility rates, and unmet needs for contraception are also critical contributors to bad maternal and child health in Pakistan [6,7]. Various strategies are proposed to improve mother–child health, including enhancing maternal health education, nutritional counseling, the participation of opinion leaders in health care programs, and mass media [8,9]. The efficacy of each method depends on the ground realities of a country or a region, as the outcomes of these strategies remained a mixed bag.

There was recent interest in maternal–child health in the period of 1990 to 2015, i.e., the time period for the achievement of MDGs has ended, and a new era of Sustainable Development Goals (SDGs) has started, of which SDG 3 is assigned for maternal child health. One study pointed out that if the same rate of child mortality prevails, 94 million children under five years of age will die. However, if these countries achieve the SDG, they can save 38 million children [10]. The global health community, therefore, needs new and improved strategies to meet the SDG. Therefore, it is necessary to find lacunas in the existing health programs that could help identify and thus provide solutions to health problems.

Pakistan, like other developing countries, lies in the lower strata of performance. According to a United National Development Program (UNDP) report, only nine out of 33 indicators have achieved their targets, while 24 indicators are off track [3]. In recent years, Pakistan took several steps to improve the health sector. For example, there are multiple health reforms for improving mother and child health care services by offering 24/7 safe deliveries in rural areas, improving vaccine cold chain management, and implementing a multi-sectoral malnutrition strategy to eliminate malnutrition [11,12].

Most of the health improvement programs are implemented in the form of projects. Projects are considered more efficient in achieving their objectives as compared to the rigid bureaucratic style of public management [13,14]. Health projects are similar to other projects, with their life cycles divided into several phases. As described above, most of the literature is focused on individual interventions; however, it is ironic that no attention has been paid to the environment in which these projects operate [15,16], i.e., the project environment where people and resources interact to achieve their goals [1,13,17]. Although several studies have focused on MNCH projects, little attention has been paid to studying the effective implementation of MNCH projects across their various phases. One such study that closely analyzed the health project is by Adebajo, Okereke, and Joseph [17], who focused on the nurses, midwives, and community health extension workers, who are involved in the provision of health care services. Similarly, Bigirwa [8] also highlighted the importance of community health workers (CHWs) in preventative and curative MNCH interventions and identified factors affecting their performance. Moreover, Perry, et al. [18] highlighted the effectiveness of community–based primary health care (CBPHC) in improving maternal, neonatal, and child health, and highlighted the importance of working closely with key stakeholders, i.e., the community that could enhance the effectiveness of such programs. However, although these studies highlighted the importance of key stakeholders, such as workers and the community, they ignored another key stakeholder, i.e., the managers of such projects. Moreover, no study has comprehensively analyzed those projects over their life cycle as to how individuals working in those projects face problems during the project life cycle and, in turn, affect the performance of the project [15,19]. The current study tries to fill this gap by delineating each phase of the National Maternal, Newborn, and Child Health (MNCH) program and describing the extent to which the existing structure works. This approach has an advantage as it helps in understanding details at the micro-level. The project comprises different phases, and each phase’s requirements are unique. Therefore, we look at each project phase from the project managers’ perspective by identifying problems and, later on, strategies at each project level are proposed.

This study analyzes the MNCH program at each project phase and delineates the issues and provides strategy at each project phase. This study therefore fills a gap by analyzing the perceptions of key stakeholders i.e., project managers at each stage of the project life cycle who worked on the national MNCH program implemented as a project in the province of Punjab in Pakistan.

## 2. Materials and Methods

This is a qualitative study using semi-structured interviews with twelve project managers of the National Maternal, Newborn, and Child Health (MNCH) program. A semi-structured interview method was followed because it allows for an in-depth analysis of the issue. Since scarce attention is paid to this area, this method provides greater scope to assess managers’ experiences [20,21] and, at the same time, allows for understanding the project implementation in an unexplored area.

We conducted interviews with district managers in the province of Punjab. There are thirty-six districts in Punjab. In each district, a district coordinator manages the project at the district level (district coordinators will be referred to as project managers from now on). At the same time, four project manager positions were vacant at the time of research. Therefore, thirty-two project managers were serving at the time of research. For our purpose, we selected only those project managers who have five or more years of experience. Such experience is important because less experienced managers may have worked in only a particular phase of a project and thus lack the holistic experience of all the necessary phases of the project, which are necessarily integrated. We were left with twelve project managers. So, we conducted twelve interviews with those respondents. We requested their prior consent through letters. After obtaining their consent, interviews were conducted. Since all the managers consented for the interview, our response rate is 100 percent. Half of the participants were aged between 30 and 40 years, while others were between 40 and 50 years. No female participants were included in the interview. It is important to understand that the MNCH program did not have any female program managers at the time of interview. Only the participant and interviewers were present in the interview, and the interview was performed only once. The demographic information of the participants (number of participants, (*n*) is 32) is provided in Table 1.

The principal interviewer had an academic background in project management with professional experience in the health sector. All interviews took place at the project managers’ offices. Respondents were facilitated by allowing them to choose the place and time of their choice. Before interviewing, respondents were informed about the research purpose. The average time of the interview was 72 min, ranging from 55 to 84 min. Participants’ consent was obtained for taking notes during the interviews. All of the interviews were conducted by I.M., while S.H. assisted him. I.M. was a master’s student at the time of interview and has a specialized background in project management, along with experience in health management and data analysis. S.H. has a specialized background in project management, along with experience in qualitative data analysis. After performing the interviews, we analyzed the data. For thematic analysis, all the necessary steps of data familiarization, generation of initial codes, and searching for the themes, their analysis, and their definition, were performed [20]. Field notes and reflections were completed after each of the interviews. M.I.M., F.S. and I.M. coded the data, while other team members also helped in the coding process. All the team members had discussions at various stages of the coding and development of themes to refine the data analysis.

Thematic analysis was chosen to analyze the data collected through interviews. This method is appropriate for the research purpose as it identifies the patterns across the data; besides, it helps in identifying the social processes in practice. Questions were revised to clarify and record relevant information, and field notes were recorded after each interview. All the interviews were recorded with the respondents’ permission. After that, interviews were transcribed and coded. Themes were identified, analyzed, and developed by all the team members.

**Figure 1:**
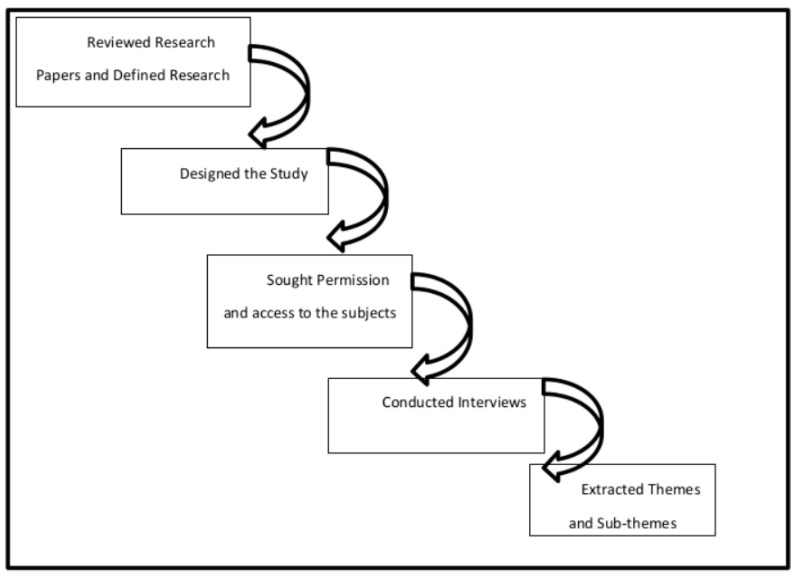
The response generation procedure.

Moreover, the ethical review process was properly adopted by sending a formal application to the head of the committee containing the title, description, and the details about the participants/respondents. The committee approved the project on after reviewing the proposal (CUIDMS/EC/CERT/090211/2019 approved on 12 May 2019). Following procedure as highlighted in Figure 1 is adopted for response generation.

## 3. Results

For the thematic analysis, we proceeded according to the project phases. Major themes were identified, which were then divided into sub-themes. Project phases were divided into six parts, and each phase was described with a major theme, which was then divided into sub-themes (see Table 2). To avoid bias in the thematic analysis and to present objective analysis, various steps were taken. First, all the team members coded the data. Although initial coding was performed primarily by two team members, other members also helped in initiating the codes. Second, we reported back the results of the analysis to the participants and, after taking their feedback, we made several minor modifications to our analysis. Third, we checked for alternative explanations and also sought help from peers, and their feedback was incorporated and various modifications were made.

Moreover, for thematic analysis, all the necessary steps of data familiarization, generation of initial codes, and searching for the themes, their analysis, and their definition, were performed.

## 4. Discussion

### 4.1. Project Phase I—Project Definition and Initiation

In the first phase of the project, the following major theme was identified, which was then divided into five sub-themes.

#### Major Theme 1—Bureaucratic Structure

- Sub-theme 1—Top-down approach
  The top-down approach is a managerial approaches where decision-making and command and control remain at the top. This approach is usually followed in health projects. In Pakistan, the projects are initiated by the federal government, relegated to provincial governments, and implemented by bureaucracy. It is ironic that during the project conception phase, there is no involvement of the project implementation teams. Non-involvement slows down the implementation process due to inadequate knowledge. As one of our respondents stated:
  “Our project was first conceived at the federal level by the Ministry of Health with due involvement of donor agencies, and a federal PC-1 (Planning Commission Form 1) (2006-11) was implemented to provinces. After that, the PC-1 is sent to the provincial governments, and the provinces implement the same in districts. However, the health administration at the district level was not involved in any stage” (PM-1(Project managers who were interviewed were given numbers thus PM 1 denotes manager who was given number 1).
- Sub-theme 2—One size does not fit all
  Health projects are initiated with the utmost care after considerable research and thought. However, these plans run the risk of trying to replicate the successful experience at another place or situation [22,23], which may not work everywhere. Socio-economic and demographic conditions and cultural values may differ considerably over different regions, even within a single country. Therefore, one approach, which has been delivered in one place, may not work in others. As one of our respondents added:
  “We cannot compare the health services provided in Lahore with the services being provided in southern districts of Punjab because the demographical conditions of districts differ because of literacy rate, per capita income and living standards.” (PM-3)
- Sub-theme 3—Financial dependency
  Health projects are highly dependent on foreign aid. Although foreign aid helps in eradicating health problems, at the same time, dependency results in adopting the practices that foreigners consider preferable, which may not work in local conditions. As recipients of the aid, locals may not express their apprehensions, which may result in the program working differently from what would have been best according to the ground realities if locals were given more authority to implement their approach [21,22,23,24]. As another respondent added:
  “Community Midwives model was implemented across the country with the support of international donor agencies by considering the high ratio of home deliveries which is nearly about 60%, but according to recent statistics, most of CMWs have failed to survive in their community, majority of them left the program and now are doing private jobs” (PM-3).
- Sub-theme 4—Lack of ownership
  The project implementation team and key stakeholders are not involved in the “project initiation phase.” The project team, as well as the key stakeholders, for example, the administrative head of the district, district finance and planning department, district accounts office, and work and services department, were completely unaware about the project and its processes, which led to administrative delays, late approvals, and ultimately resulted in a higher than expected cost. One of the respondents added:
  “Under the National MNCH project, we were given the responsibility to construct a training school for community midwives at an estimated (Pakistani rupees) Rs cost. 62.5 million in PC-1 during the financial year 2007–2008. However, when we have to implement that project, neither Ministry of Health nor the finance or public works department owns it. All these stakeholders were waiting for the project money to be released. When money was released, it was too late, and the project, which was supposed to be delivered in 2008–2009, was completed in 2011–2012 at Rs. 90 million. I think the reason being that neither we were involved at the time of planning, nor we had any idea how we would do it. I also know similar kinds of projects in other districts that are still incomplete” (PM-6).
- Sub-theme 5—Lack of awareness
  It has been observed that the project implementation team was not provided with any orientation about the project. Even the essential documents (for example, PC-1) were not shared with them. Therefore, they were not aware of the primary objectives of the project. As one of our respondents stated:
  “It has been six years. I am working with the National MNCH project, and I never had any orientation about the project nor know its key objectives. I simply learn things by doing, because I receive orders from higher authorities in parts (i.e., overtime)” (PM-7).

### 4.2. Project Phase II—Project Planning

The following major themes were developed during this phase:

#### Major Theme 2—Lack of Realistic Planning

- Sub-theme 1—Unreliable data
  Planning a project is the most important part of any project during the planning phase. The most critical issue is the reliability of the data. As all the planning depends on the data, any problem in the data may jeopardize planning. During the course of the interviews, our respondents highlighted reliability issues in the data based on which future planning is being done. As one of the project managers added:
  “I have great concern over the reliability of data on which planning is being done. For example, as per MDGs, safe deliveries by skilled birth attendants (SBA) should reduce maternal mortality rate (MMR). After the promulgation of Chief Minister Health reforms back in 2013, safe deliveries by SBA has increased by eight folds, which should have reduced the MMR significantly, but I do not see any changes at all in the MMR, and I seriously doubt the reliability of the data (PM-1)”.
- Sub-theme—2 Unrealistic targets
  It is also important to note that the government, to make things go according to the plan, considers it important to achieve its objectives by assigning targets. Achievement of targets will ultimately result in the achievement of objectives. However, the problem lies in the context under which these targets were to be achieved. For example, one of our respondents highlighted:
  “The main reason behind poor outcome is the allocation of unrealistic targets assigned from federal/provincial governments without considering the areas’ demographic condition. For example, we cannot compare District Lahore with District Chakwal in any aspect, but both districts are given almost the same targets. Therefore, when targets are assigned to field staff, they may tend to manipulate the data” (PM-4).

### 4.3. Project Phase III—Project Implementation

The following theme was developed during the third phase:

#### Major Theme 3—Lack of Sustainable Working Environment

- Sub-theme 1—High employee turnover
  For the smooth running of the project, it is important to have a sustainable working environment. The sustainable environment, in turn, requires that project employees, especially at the senior level, should be associated with the project until its completion. During the course of the interviews, we noted high employee turnover at the top level of management, as one of our respondents added:
  “Projects are run by senior bureaucrats. I have two major observations. Firstly, they (project managers) lack in professional project skills. Secondly, only those bureaucrats are assigned as project managers near the age of retirement. Even of those managers, when they get the necessary know-how during their tenure, either they get transferred to other departments or get retired; the newly appointed one has to start from scratch. For example, during the last three years, we had four different project managers in our project” (PM-1)
- Sub-theme 2—Rigid structures
  It is evident from the above discussion that most of the project managers do not have project management skills that are necessary to execute a project to avoid negative outcomes [7,8,9,10]. Most of them are used to the existing “Weberian” rigid bureaucratic structure. Thus they try to manage the projects as they have learned over their years of service in a bureaucratic structure. This practice creates problems, as one of our respondents noted:
  “The working structure of existing public projects is very different from the universally accepted practices of project management. Higher authorities appoint us, and we have to run the project similarly as desired by them” (PM-9).
- Sub-theme 3—Lack of continuous funding
  Besides, for smooth working, the project needs timely financial resources [21,25]. However, in Pakistan, currently, projects are financed over a quarterly basis, and even that financing is usually delayed. A respondent added:
  “Our financial grants are not regular; we are never asked about our financial requirements, and the grants which are issued from the province are always delayed, mostly we do not receive the financial grant in time, while we have a long list of pending liabilities waiting for those finances. In some cases, finances are not provided at all”. (PM-9)
- Sub-theme 4—Lack project management techniques
  Various methods help in project management. As project managers, one has to know about these techniques, including knowledge of some software programs. However, our respondents seem to be completely unaware of such developments. As one of the respondents stated:
  “We are medical professionals and not the project management or IT experts, nor have we given any training on the use of such tools and techniques to improve management of the project” (PM-3).

### 4.4. Project Phase IV—Project Monitoring and Control

The following theme was developed during the fourth phase:

#### Major Theme 1—Lack of Authority and Political Interference

- Sub-theme 1—Lack of authority
  Poor control over project resources leads to poor project results. The MNCH project is implemented at the district level. Being part of the district administrative system, managers have to comply with district authorities’ instructions. Therefore, project managers are under pressure to obey district authority’s instructions, even if some of their orders are not legal. These orders are usually about the project resources that the district administration use for their own purpose. As one of the respondents stated:
  “We have to share our project resources with others (local administration) for uses other than the MNCH project, as we have minimal authority and control over administrative matters and project resources. We have to compromise over inappropriate use of project resources “(PM-2).
- Sub-theme 2—Political involvement
  There is also political interference in the projects that interferes with the project portfolio management [6,24]. This interference comes in two ways: firstly, the project managers have to appoint incompetent employees because of undue recommendations of the political elite. Secondly, disciplinary action against such employees is tough to exercise because of the politicians’ support. As one of the respondents stated:
  “We are usually under pressure to employ their (politicians’) people, and whenever we try to take any disciplinary action against some of our employees, local politicians put pressure against that action” (PM-6).

### 4.5. Project Phase V—Closing the Project

#### Major Theme 1—No Lesson Learned

One of the essential aspects of project management is what we have learned during the project, as these lessons increase our knowledge. These lessons can be used in similar types of projects, and one would be able to use the best practices learned during the project and avoid traps that could harm similar projects. Such practice is non-existent in the MNCH project, as one of the respondents simply denied the existence of such practices by answering:

“No lesson learned document is prepared after completion of the approved period of PC-1 at district level” (PM-7).

We have identified various themes that emerged from the interviews. These themes helped to identify various problems faced by the MNCH project in Pakistan. As stated earlier, there is a dearth of studies that analyze health projects and pinpoint why those projects failed [25,26], specifically in the public sector. Although we analyzed a single project, our results could also apply to many health projects, especially in developing countries. The following recommendations are made based on policy gaps identified.

The traditional bureaucratic structure may not work in projects; there is a need to incorporate input from the community level in the project initiation phase. The top-down approach can be replaced by the bottom-up approach as it is considered most suitable whenever service delivery is required. Furthermore, incorporating input from all key stakeholders, with emphasis on region-specific variables like socio-economic conditions and demographic peculiarities, can contribute to better policy-making.

While initiating the project, central decision-making and a lack of awareness about the ground realities inhibit progress. It is suggested that the project implementation managers should be involved while defining the project charter.

Planning for a project requires reliable data and the involvement of project managers as well as their teams. We propose a third party check on the data reliability. We also propose the concept of whistleblowing in the health projects [26,27]. Targets should be realistic and be region specific, based on demographic data. Regular feedback should be communicated to all stakeholders for the incorporation of the project implementation team’s recommendation in future planning. The central part of the project is its execution, which can be improved by having a sustainable and flexible work environment, motivated employees, and a continuous flow of resources while having competent project managers with full authority. We propose that there should be stability in the tenure of employees. Project financing should be smooth and regular. We propose the hiring of professional project managers to supplement the working of projects, as well as training existing project managers in the latest project management techniques.

Projects needs continuous monitoring and evaluation as they proceeds. They requires constant checks on the project progress. We propose that project managers should have the authority to make decisions about accountability issues. A transparent chain of authority with a single command should be established. To minimize political interference, we propose the introduction of the e-monitoring system to improve surveillance. Similarly, we propose the introduction of a patient feedback system at the community level. The project manager should have the authority to acquire project resources.

## 5. Conclusions

This study found that project managers experience various problems from the initiation stage of the project to its closure. Additionally, they found themselves to be poorly equipped to manage such problems. Most of them talked about the problems. However, a rigid bureaucratic structure and strict compliance to the authoritarian style left them with little choice but to follow the orders, while contributing little from their side. However, one aspect they talked about was their inability to understand project management techniques because of having almost no background in project management, which reduced their ability to add to the project. Both these problems left them with little ability to make innovative decisions according to the situation.

Besides the problems at the individual project manager’s level, they also had to face systemic problems. They often faced political pressure, a lack of financial resources, and little authority over the use of available resources. This state of affairs made them feel helpless in the face of new challenges. These problems were exacerbated by being provided with unreliable data and unrealistic targets that, in some cases, were very hard and even impossible to achieve. In this situation, there was a danger of having a strong urge on the part of project managers to portray a rosy picture of the project, thereby further compromising the accuracy of the data reported.

Even after completing the project, one needs to learn from his/her experiences in the project. There should be a proper system for the submission of project progress reports and project learning reports.

## Figures and Tables

**Table 1:** Information about the respondents, *n* = 32.

Variable	Category	Frequency	Percentage
Gender	Male	32	100.0
	Female	00	00.0
Age (years)	26–35	08	25.0
	36–45	17	53.1
	46–55	07	21.8
Education	Graduate	12	37.5
	Master’s	20	62.5
Experience (years)	<1	Nil	00.0
	1–5	Nil	00.0
	6–10	11	34.3
	11–15	12	37.5
	<15	09	28.1

Source: Field data. *n* = number of participants.

**Table 2:** Themes and sub-themes.

Themes	Short Description
Project Phase I—Project definition and Initiation	
	Major Theme 1—Bureaucratic structure
	Sub-theme 1—Top-down approach
	Sub-theme 2—One size does not fit all
	Sub-theme 3—Financial dependency
	Sub-theme 4—Lack of ownership
	Sub-theme 5—Lack of awareness
Project Phase II—Project Planning	
	Major Theme 2—Lack of realistic planning
	Sub-theme 1—Unreliable data
	Sub-theme 2—Unrealistic targets
Project Phase III—Project Implementation	
	Major Theme 3—Lack of sustainable working environment
	Sub-theme 1—High employee turnover
	Sub-theme 2—Rigid structures
	Sub-theme 3—Lack of continuous funding
	Sub-theme 4—Lack project management techniques
Project Phase IV—Project Monitoring and Control:	
	Major Theme 1—Lack of authority and political interference
	Sub-theme 1—Lack of authority
	Sub-theme 2—Political involvement
Project Phase V—Closing the Project	
	Major Theme 1—No lesson learned
